# microRNA-99a is downregulated and promotes proliferation, migration and invasion in non-small cell lung cancer A549 and H1299 cells

**DOI:** 10.3892/ol.2015.2873

**Published:** 2015-01-14

**Authors:** CHANGJIN CHEN, ZIYI ZHAO, YU LIU, DEZHI MU

**Affiliations:** 1 Department of Pediatrics, West China Second University Hospital, Sichuan University, Chengdu, Sichuan 610041, P.R. China; 2 Center Laboratory, Teaching Hospital of Chengdu University of TCM, Chengdu, Sichuan 610072, P.R. China; 3 Key Laboratory of Bio-resources and Eco-environment (Ministry of Education), College of Life Sciences, Sichuan University, Chengdu, Sichuan 610041, P.R. China

**Keywords:** microRNA-99a, insulin-like growth factor 1 receptor, non-small cell lung cancer, diagnosis, epithelial to mesenchymal transition

## Abstract

There is increasing evidence that microRNAs (miRNAs) are able to play a key role in the diagnosis and therapy of cancer. miRNA-99a (miR-99a), which is downregulated in several human malignancies, has been reported as a potential tumor suppressor. However, to the best of our knowledge, the expression and function of miR-99a has not been investigated in human non-small cell lung cancer (NSCLC) at present. The aim of the current study was to evaluate the association between NSCLC and miR-99a. miR-99a expression was analyzed in 15 pairs of NSCLC and non-cancerous tissue samples by reverse transcription-quantitative polymerase chain reaction. In addition, the NSCLC A549 and H1299 cell lines were transfected with miR-99a mimics, and the effect of miR-99a on the cell cycle, cell proliferation, migration and colony formation of A549 and H1299 cells was investigated. It was found that the level of miR-99a expression was significantly downregulated in NSCLC tissues and that ectopic overexpression of miR-99a significantly inhibited the growth of A549 and H1299 cells. Additionally, ectopic overexpression of miR-99a inhibited A549 and H1299 cell migration and invasion by inhibiting epithelial to mesenchymal transition. The downregulation of insulin-like growth factor 1 receptor (IGF-1R) by miR-99a and knockdown of IGF-1R mediated by siRNA were each found to phenocopy the effect of miR-99a overexpression in NSCLC. To the best of our knowledge, the present study demonstrated for the first time that, in NSCLC, miR-99a is downregulated and thus regulates proliferation, colony formation and migration through the IGF-1R pathway, which indicates that miR-99a is a diagnostic biomarker for NSCLC.

## Introduction

Non-small cell lung cancer (NSCLC), which includes adenocarcinoma and squamous cell carcinoma, is the predominant form of lung cancer and remains the leading cause of cancer-associated mortality in the world, particularly in China (1,2), accounting for >80% of all cases of lung cancer. It is hypothesized that NSCLC will continue to be a major health hurdle for mankind over the next century (3). NSCLC tumorigenesis is not only strongly associated with certain protein-coding genes, including p53, Rb and Ras (4), but is also regulated by ~100 microRNAs (miRNAs) (5). Therefore, an improved understanding of the molecular mechanisms regulated by microRNAs involved in NSCLC development is required as a basis to identify novel strategies for the treatment of these diseases.

miRNAs are a class of endogenous non-coding RNAs, ~20–25 nucleotides long, that are widely expressed in eukaryotes and predominantly inhibit gene expression at the post-transcriptional level by base pairing with target mRNAs in the 3′-untranslated region, leading to mRNA cleavage or translation repression (6–8). The levels of individual miRNAs vary significantly between tissues, developmental stages and physiological processes, indicating that miRNAs play a role in cellular proliferation and differentiation, tumorigenesis and apoptosis (9,10). Deregulation or mutation of miRNAs has been frequently reported in numerous human malignancies, including breast carcinoma, primary glioblastoma, lung cancer and colon carcinoma (11–14), and has been found to contribute to the initiation and progression of cancer (15). miRNA is able to function as either a tumor suppressor or an oncogene (16). The tissue- and disease-specific expression patterns of miRNAs indicate their potential as diagnostic and prognostic cancer biomarkers and therapeutic tools (17).

miR-99a, which is transcribed from the 21q21 region, has been reported to be deregulated in renal cell carcinoma (RCC) (18). miR-99a induces G_1_-phase cell cycle arrest and suppresses tumorigenicity, functioning as a tumor suppressor in RCC. It has also been reported that, in NSCLC, the miR-99a gene locates to a homozygous deletion region, indicating that miR-99a may play a crucial role in tumorigenesis and cancer progression (5). miR-99a has been reported to be downregulated in squamous cell lung carcinoma (19). However, to the best of our knowledge, there have been no studies investigating the role of miR-99a in adenocarcinoma.

In the present study, the expression of miR-99a in adenocarcinoma tissues was examined, and the impact of miR-99a on A549 and H1299 NSCLC cells was assessed.

## Materials and methods

### Tissue samples and cell lines

Paired NSCLC and normal adjacent lung tissues were obtained, with informed consent, from 15 patients who underwent primary surgical resection of NSCLC between 2011 and 2012 at the Teaching Hospital of Chengdu University of Traditional Chinese Medicine (Chengdu, Sichuan, China). The NSCLC A549 and H1299 cell lines, and the normal lung HBE cell line were frozen in the Functional Genomic Laboratory, Sichuan University (Chengdu, Sichuan). The HBE and A549 cells were maintained in Dulbecco’s modified Eagle’s medium (DMEM; Invitrogen, Carlsbad, CA, USA), and the H1299 cells were maintained in RPMI-1640 medium, each supplemented with 2 mM L-alanyl-L-glutamine, 1% penicillin/streptomycin and 10% fetal bovine serum (Invitrogen) at 37°C and 5% CO_2_.

### RNA extraction and stem-loop conventional reverse *transcription-polymerase* chain reaction (RT-PCR) analysis

Total RNA was isolated using TRIzol reagent (Invitrogen) according to the manufacturer’s instructions. Reverse-transcribed complementary DNA was synthesized using the Prime-Script RT reagent kit (Takara Biotechnology Co., Ltd., Dalian, China). Conventional PCR was used to assay miRNA expression with the specific forward primers, and the universal reverse primer complementary to the anchor primer and U6 small nuclear RNA was used as the internal control. The PCR primers for mature miR-99a or U6 were designed as follows: miR-99a forward, 5′-ACAGTCGAGATGGGATAC CCTTACCATTACT-3′ and reverse, 5′-CTGCTGACGTCGA GTGGGCAA-3′; and U6 forward, 5′-CTCGCTTCGGCAGCA CA-3′ and reverse, 5′-AACGCTTCACGAATTTGCGT-3′. The PCR cycles were performed by initial denaturation at 95°C for 5 min, then by completing 40 cycles at 95°C for 10 sec followed by 60°C for 1 min.

### Plasmid construction and miRNA transfection

The plasmids pMSCV-miR-99a and pMSCV-miR-NC were kindly provided by Dr R Agami (Faculty of Science, Ain Shams University, Cairo, Egypt) (20). Stable transfection of pMSCV-miR-99a resulted in mock A549 (A549-miR-99a) and mock H1299 (H1299-miR-99a The 2′-O-methyl oligonucleotides were chemically synthesized by LifeTechnologies (Guangzhou, Guangdong, China). The oligonucleotide sequences were as follows: miR-99a mimic forward, 5′-AACCCGUAGAUCCGA UCUUGUG-3′ and reverse, 5′-CAAGAUCGGAUCUACGGG UUUU-3′; miR-negative control (miR-NC) forward, 5′-UUC UCCGAACGUGUCACGUTT-3′ and reverse, 5′-ACGUGAC ACGUUCGGAGAATT-3′. The A549 (5×10^5^) and H1299 (3×10^5^) cells were seeded 24 h prior to 48-h transfection with the miR-99a mimic or miR-NC, respectively. The transfections were performed using Lipofectamine 2000 (Invitrogen) according to the manufacturer’s instructions. The cells were harvested for further testing 48 h after transfection.

### Cell proliferation assay

Cell proliferation was detected using a 3-(4, 5-dimethylthazol-2-yl)-2, 5-diphenyltetrazolium bromide (MTT) assay. The cells were seeded into 24-well plates (1.2×10^4^ cells/well) and allowed to attach overnight. After 24, 48, 72 and 96 h, cell viability was assessed using an MTT assay. The absorbance at 490 nm of each well was read on a spectrophotometer. Three independent experiments were performed in quadruplicate.

### Colony formation assay

In total, ~5×10^3^ cells from each group, mock A549 (A549-miR-99a), stably transfected A549 (A549-miR-NC), mock H1299 (H1299-miR-99a) and stably transfected H1299 (H1299-miR-NC) cells, were placed in a six-well plate containing RPMI-1640 medium supplemented with 10% FBS for three weeks. The colonies were fixed with methanol and stained with 0.1% crystal violet (Sheng Gong, Shanghai, China) in 20% methanol for 30 min. Each assay was performed in triplicate.

### Cell cycle assay

Transfected A549 and H1299 cells in the log phase of growth were collected and fixed in 75% ethanol at −20°C for 16 h. For the cell cycle analysis, the transfected cells were stained with propidium iodide (PI) and examined with a fluorescence-activated cell sorting (FACS) flow cytometer (BD Biosciences, San Jose, CA, USA). Each test was performed in triplicate.

### Cell migration and invasion assay

The migratory and invasive potential of the transient transferred cells and bulk-selected A549 and H1299 cells were examined. A scratch assay was performed to assess the migratory potential. The cells were scratched using a pipette tip when the cell confluence reached ~95%, and were further incubated with fresh medium. The medium was changed every two days. Images were captured at a ×40 magnification immediately subsequent to scratching (0 h) and at 12 and 24 h subsequent to scratching. The cell invasion assay was tested using Transwell plates (8-μm pore size; 6.5-mm diameter; Corning Life Sciences, Tewksbury, MA, USA) pre-coated with Matrigel Basement Membrane Matrix and at a concentration of 1 mg/ml (BD Biosciences, Franklin Lakes, NJ, USA), according to the manufacturer’s instructions. In total, 2×10^4^ cells in 0.2 ml media, supplemented with 2% FBS, were seeded into the upper chamber, with 0.6 ml of medium containing 10% FBS under the upper chamber. The plates were incubated at 37°C in a 5% CO_2_ atmosphere. After 48 h, the chambers were removed and a cotton swab was used to remove the non-invading cells from the upper side of the chamber. The cells under the chamber were then fixed in methanol for 10 min and stained with 0.1% crystal violet in 20% methanol for 30 min.

### Western blot assay

Protein extract (50 μg) was separated by 10% SDS-polyacrylamide gel electrophoresis (SDS-PAGE) and was electrophoretically transferred onto a polyvinylidene fluoride membrane (Millipore, Darmstadt, Germany). The membranes were blocked for 30 min at room temperature with 5% non-fat dried milk and incubated for 1 h with monoclonal antibodies against E-cadherin (sc-8426, mouse, IgG_1_), N-cadherin (sc-7939, rabbit, IgG), γ-catenin (sc-30997, goat, IgG), insulin-like growth factor 1 receptor (IGF-1R; sc-sc462, mouse, IgG_1_) or β-actin (sc-1616, goat, IgG) (Santa Cruz Biotechnology, Dallas, TX, USA). The antibodies against E-cadherin, N-cadherin and γ-catenin were at a dilution of 1:1,000 and the antibody against β-actin was at a dilution of 1:5,000. Subsequent to washing with PBS-T (10 mm Tris, pH 8.0; 150 mm NaCl; 0.5% Tween 20; Sheng Gong), the membranes were incubated for 1 h with secondary HRP-linked polyclonal goat anti-rabbit antibody (sc-2004, IgG, Santa Cruz Biotechnology), at a 1:20,000 dilution. The membranes were washed again with PBS-T and the proteins were visualized using ECL chemiluminescence and exposed to X-ray film.

### Statistics

Statistical analyses were performed using the GraphPad prism software (GraphPad Software, Inc., La Jolla, CA, USA). Statistical differences were calculated using an unpaired two-tailed student’s t-test. P≤0.05 was considered to indicate a statistically significant difference. The statistical significance of a correlation was assessed using the Pearson test.

## Results

### miR-99a is significantly downregulated in human NSCLC tissues

**Figure 1:**
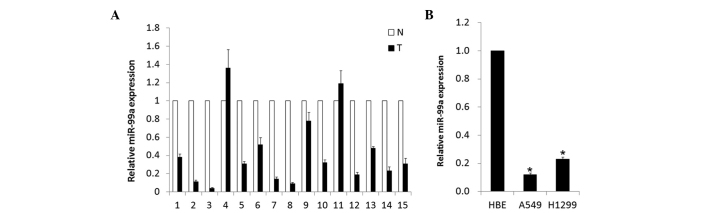
Relative expressions of miR-99a in NSCLC tissues and various NSCLC cell lines by RT-qPCR. (A) miR-99a expression profile in NSCLC tissues compared with normal adjacent tissues. (B) Relatvie expressions of miR-99a in different NSCLC cell lines by RT-qPCR. The relative expression values were calculated using the equation relative quantification = 2^−ΔΔCT^. ^*^P<0.05. NSCLC, non-small cell lung cancer; RT-qPCR, reverse transcription-quantitative polymerase chain reaction; miR-99a, microRNA-99a; N, normal tissue; T, tumor tissue.

In the present study, a stem-loop RT-quantitative PCR assay was performed to determine the expression of miR-99a in 15 pairs of matched NSCLC and normal adjacent lung tissues. As shown in Fig. 1A, the miR-99a expression levels were all significantly reduced by 2.1–25 times in 86.7% (13/15) of NSCLC tumor tissues compared with the corresponding adjacent normal lung tissues. Of the 15 tested lung cancer tissues, the expression level of miR-99a demonstrated no difference between ages, genders or metastasis status. To further assess the biological role of miR-99a in adenocarcinoma cell lines, its expression level was detected in A549 and H1299 cells. Compared with the normal human bronchial epithelial HBE cell line, the level of miR-99a was markedly decreased (Fig. 1B). These results revealed that the expression of miR-99a was downregulated in NSCLC adenocarcinoma tissues and cell lines, indicating an involvement in NSCLC carcinogenesis.

### miR-99a suppresses tumorigenicity by inducing G_1_-phase cell cycle arrest in vitro

**Figure 2:**
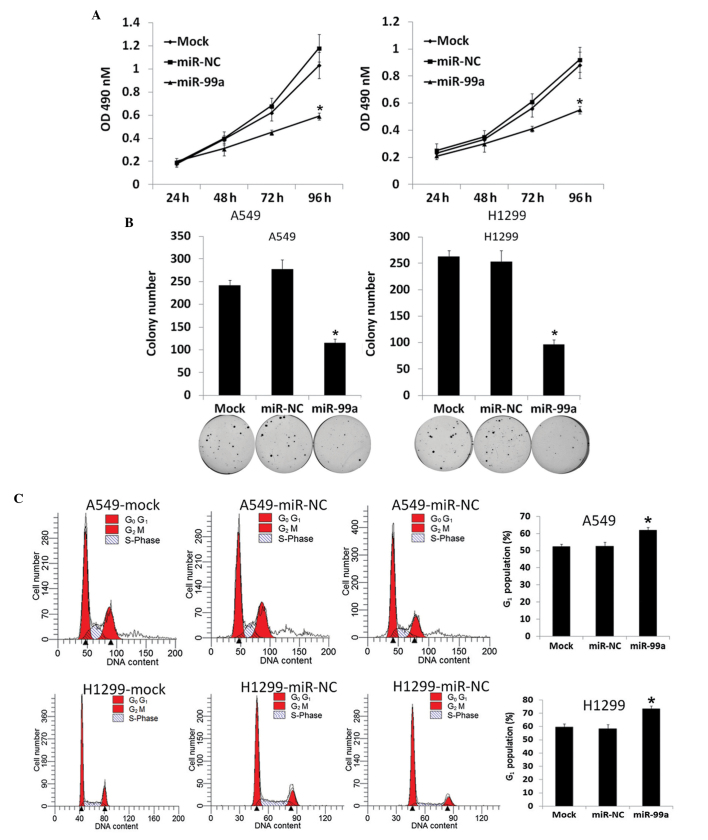
Expression of miR-99a in A549 and H1299 cells inhibits cell proliferation and colony formation by inducing G_1_-G_0_ cell cycle arrest. (A) A549 and H1299 mocks, stably transfected with miR-NC and miR-99a, were assessed using MTT assays to determine the proliferation of A549 or H1299 cells. The data are presented as the mean ± standard deviation from three independent experiments. (B) Colony formation assays were performed to determine the proliferation of A549 or H1299 cells. The colonies were counted and images of the colonies were captured. (C) Histograms for red fluorescence quantified by flow cytometry of A549 and H1299 mock cells, which stably expressed miR-NC or miR-99a. ^*^P<0.05. miR-99a, microRNA-99a; miR-NC, miR negative control; OD, optical density.

The downregulation of miR-99a in NSCLC adenocarcinoma cell lines prompted the identification of its activity as a tumor suppressor. First, miR-99a was restored in A549 and H1299 cell lines that contained a relatively low miR-99a level, and MTT, colony formation and flow cytometric assays were then performed. As shown in Fig. 2A, the A549-miR-99a and H1299-miR-99a cell lines exhibited a significant increase in cell viability compared with the mock A549 and H1299, A549-miR-NC or H1299-miR-NC cell lines (P<0.05). As shown in Fig. 2B, A549-miR-99a and H1299-miR-99a cells exhibited notably fewer and smaller colonies compared with negative controls (P<0.05). The flow cytometry analysis revealed that A549-miR-99a and H1299-miR-99a cells underwent a significant increase in the proportion of cells in the G_1_-phase population compared with control cell lines (Fig. 2C). Additionally, the effect of miR-99a on apoptosis was also detected and it was found that exogenous miR-99a did not influence apoptosis (data not shown). Thus, the upregulation of miR-99a was able to induce growth inhibition by blocking the cell cycle at the G_1_ phase.

### MiR-99a suppresses the migration and invasion of NSCLC cell lines in vitro

**Figure 3:**
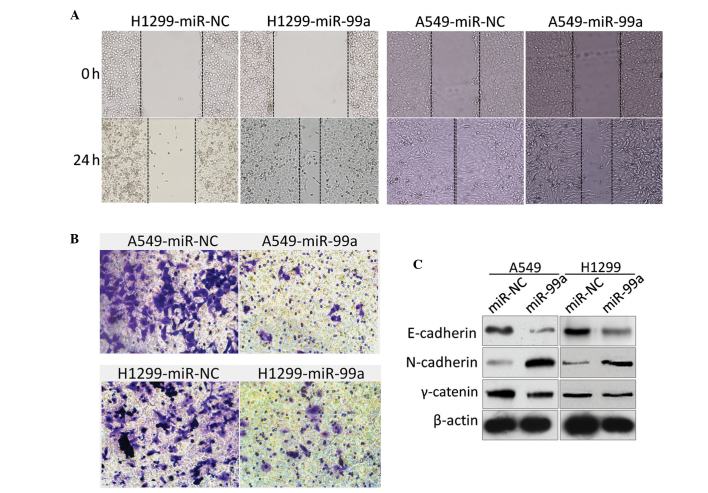
Overexpression of miR-99a inhibits the migration and invasion of non-small cell lung cancer cells. (A) The effect of miR-99a expression on cell migration was detected by a scratch assay at the indicated time-points, and also by (B) Transwell assay. (C) In the A549 and H1299 cells transfected with miR-NC or miR-99a, the protein levels of E-cadherin, N-cadherin and γ-catenin were detected by western blotting. miR-99a, microRNA-99a; miR-NC, miR negative control.

To test the role of miR-99a in the migration and invasion of NSCLC adenocarcinoma cell lines, the A549-miR-99a and H1299-miR-99a cells were analyzed by performing scratch and Transwell assays. As expected, the A549-miR-99a and H1299-miR-99a cells underwent a morphological change of cells to an elongated shape and exhibited a reduction in migration (Fig. 3A and B). Western blot analysis was then performed to detect the changes in N-cadherin, E-cadherin and γ-catenin. A corresponding marked increase in E-cadherin and γ-catenin and decrease in N-cadherin were also observed (Fig. 3C).

### MiR-99a inhibited EMT by decreasing the IGF-1R level

**Figure 4:**
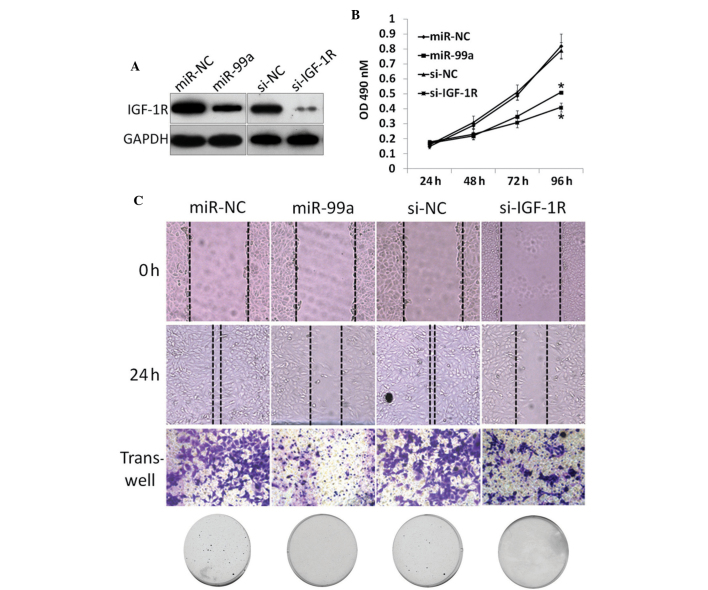
miR-99a is targeted to IGF-1R and affects non-small cell lung cancer cells by downregulating IGF-1R. (A) IGF-1R protein levels in A549-miR-99a or A549-miR-IGF-1R cells were detected by western blotting. (B) The proliferation of the cells in (A) was assessed by performing an MTT assay. (C) The colony formation, migration and invasion of these cells were then assessed. ^*^P<0.05. IGF-1R, insulin growth factor 1 receptor; miR-99a, microRNA-99a; miR-NC, miR negative control; OD, optical density; si-NC, small interfering RNA negative control; si-IGF-1R; si-insulin-like growth factor-1 receptor.

To further reveal the mechanisms underlying this tumor suppressor effect of miR-99a, IGF-1R, a target mRNA of miR-99a, was knocked-down in NSCLC cells. The A549 cells were transfected with IGF-1R siRNA or negative control (NC), followed by functional assays (Fig. 4A). Proliferation and colony formation results revealed that knockdown of IGF-1R decreases proliferation (Fig. 4B) and colony formation (Fig. 4C), similar to the phenotype observed upon miR-99a restoration in the A549 and H1299 cells. Scratch and Transwell assays also revealed a similar phenotype compared with miR-99a restoration (Fig. 4C). Collectively, it was concluded that the tumor suppressor role of miR-99a is associated with IGF-1R pathway regulation.

## Discussion

Lung cancer is considered to be the most dangerous cancer worldwide and is a leading cause of cancer mortality. Currently, a model for lung cancer pathogenesis hypothesizes that the pathogenesis is due to the combination of genetic risk and environmental factors (20). As aforementioned, NSCLC accounts for ~80% of all lung cancer subtypes (3). Understanding the carcinogenic mechanisms may aid in the identification of a biomarker or therapeutic target for lung cancer, and this promotes the elucidation of the underlying mechanism of NSCLC development. Although the carcinogenesis and pathophysiology of NSCLC have been intensively investigated in the past several decades (1,2), the underlying mechanism of NSCLC development remains poorly understood and no efficient therapeutic strategies have emerged from the extensive number of studies that have been performed.

As a prevailing area for cancer biology studies in previous years, miRNA has been revealed to play important regulatory roles in tumorigenesis and cancer development, not only in cell proliferation and development (21–23). The regulatory roles of miRNA include the upregulation of mir574-3p in prostate cancer (24) and the extreme downregulation of miR-23a in gastric cancer (25). The miRNAs that have been identified as associated with lung cancer include miR-210 (26), miR-365 (27) and miR-449 (28), indicating an association between miRNA and NSCLC.

In the present study, it was found that miR-99a was significantly downregulated in NSCLC adenocarcinoma tissues and cell lines, which was consistent with previous findings that miR-99a is reduced in several human tumors and cancer cell lines compared with normal adjacent lung tissues and normal cell lines (19). The expression of miR-99a was low in 13 out of 15 NSCLC tissues, although no correlation was observed between the reduction in miR-99a expression and the clinical characteristics of NSCLC in the 15 pairs of tissues. This indicates that low expression of miR-99a may be involved in NSCLC carcinogenesis. In addition, it was demonstrated that miR-99a expression is elevated in NSCLC cell lines compared with the HBE normal human bronchial epithelial cell line, with the exception of A549 and H1299, and these findings also indicate the role of miR-99a in NSCLC adenocarcinoma carcinogenesis.

To assess the role of miR-99a in NSCLC, the present study investigated the effect of miR-99a gain of function on various aspects of NSCLC. First, it was demonstrated that the overexpression of miR-99a in A549 and H1299 cells, two adenocarcinoma cell lines, led to a significant inhibition of cell proliferation, colony formation, migration and invasion.

IGF-1R mRNA is an identified miR-99a target that is known to be involved in the pathogenesis of psoriasis (29,30). Although the mechanisms of IGF-1R in EMT are unclear, the downregulation of IGF-1R is sufficient to drive tumor cell migration, indicating that the presence of IGF-1R is critical for inducing an EMT-like phenotype. The present study hypothesized that miR-99a-mediated downregulation of IGF-1R induced an EMT-like phenotype. As expected, siRNA-mediated IGF-1R knockdown caused a morphological change in cells to a flatter shape. A corresponding decrease in the levels of E-cadherin and γ-catenin, and an increase of N-cadherin was also observed, which was similar to the effects of miR-99a overexpression, indicating that overexpression of miR-99a alters the epithelial phenotype of the cell in an IGF-1R-dependent manner. In addition to EMT, there is usually an increase in cell mobility. Consistent with this phenomenon, IDL single-stranded RNA expression markedly increased the cell migration of A549 cells, as examined by scratch and Transwell assays.

In conclusion, the present study identified miR-99a as an effector of the IGF-1R pathway during EMT in NSCLC cells. The data revealed that miR-99a is significantly downregulated in NSCLC adenocarcinoma cells and it can be hypothesized that miR-99a may play a key role in NSCLC development and progression by modulating IGF-1R signaling. The identification of the downregulation of miR-99a in NSCLC adenocarcinoma highlights the possibility of therapeutic applications for miR-99a in cancer.
