# [Expression of Concern] microRNA-99a is downregulated and promotes proliferation, migration and invasion in non-small cell lung cancer A549 and H1299 cells

**DOI:** 10.3892/ol.2025.15305

**Published:** 2025-10-01

**Authors:** Changjin Chen, Ziyi Zhao, Yu Liu, Dezhi Mu

Oncol Lett 9: 1128–1134, 2015; DOI: 10.3892/ol.2015.2873

Following the publication of this paper, it was drawn to the Editor's attention by a concerned reader that, regarding the scratch wound assay experiments shown in Figs. 3A and 4C, the “24 h/A549-miR-NC” panel in Fig. 3A and the “24 h/miR-NC” panel in Fig. 4C contained an overlapping section of data, suggesting that these data had been derived from the same original source; moreover, the Transwell assay data shown in Figs. 3B and 4C were strikingly similar to data that subsequently appeared in other articles written by different authors at different research institutes a few years afterwards.

The authors were contacted by the Editorial Office to offer an explanation for these apparent anomalies in the presentation of the data in this paper; however, up to this time, no response from them has been forthcoming. Owing to the fact that the Editorial Office has been made aware of potential issues surrounding the scientific integrity of this paper, we are issuing an Expression of Concern to notify readers of this potential problem while the Editorial Office continues to investigate this matter further.

